# Can the establishment of an innovative city improve the level of technological entrepreneurship?

**DOI:** 10.1371/journal.pone.0289806

**Published:** 2023-10-10

**Authors:** Ye Xu, Zhi-Chao Wang

**Affiliations:** 1 School of Statistics, Jiangxi University of Finance and Economics, Nanchang, Jiangxi, China; 2 Key Laboratory of Data Science, Jiangxi University of Finance and Economics, Nanchang, Jiangxi, China; 3 Jiangxi University of Software Professional Technology, Nanchang, Jiangxi, China; Chongqing University, CHINA

## Abstract

Based on the data of 278 prefecture-level city panels in China from 2008 to 2020, this paper presents the policy of innovative pilot city as a quasi-natural experiment. It is found that (1) the implementation of innovative urban policy can significantly improve the level of science and technology entrepreneurship, but the pilot policy has a time lag effect and has a continuous promoting effect since the third year. (2) For large cities, areas with high levels of economic development, central and eastern regions and cities with high new infrastructure, innovative cities can improve the level of technological entrepreneurship; (3) Innovative cities improve the level of technological entrepreneurship by improving the incubator of technological enterprises and strengthening the flow of human capital; (4) The effect of the establishment of innovative cities on the level of technological entrepreneurship mainly shows that the siphon effect does not show radiation effect, and this siphon effect mainly improves the level of technological entrepreneurship by increasing the number of incubators of technological enterprises.

## 1. Introduction

As a knowledge-technology-intensive activity, technology-based enterprise entrepreneurship is an important force to stimulate market vitality, increase employment opportunities, promote the transformation of innovation results and promote high-quality economic development. However, because of the complex competitive environment and entrepreneurial environment, coupled with the scarcity of scientific and technological talents and the high demand for technology, the failure rate of technological enterprises’ entrepreneurship has remained high. In 2007, our country put forward the slogan "Mass entrepreneurship, mass innovation," aimed at improving economic vitality through entrepreneurship and innovation, thereby solving the employment problem. However, with the contrast between the continuous introduction of science and technology entrepreneurship policy and the low quality of innovation and entrepreneurship, let us further realize the need to improve science and technology entrepreneurship policy system from the top-level design aspect. In addition, the high risk and high demand of technological entrepreneurship reduce people’s entrepreneurial enthusiasm to some extent, so more relevant policies are needed to improve people’s entrepreneurial enthusiasm. To this end, China implemented an innovative city policy in 2009, which aims to drive technological enterprises to start their own businesses by creating a good innovation environment. But the country has been implementing innovative city policy for so long, its policy effect, can it improve technology enterprise entrepreneurship? If it can improve its entrepreneurial level, through what path? Answering the above questions is of great significance for improving the high-quality development of our economy under the conditions of the new era.

There are three types of literature most closely related to this article: (1) Focus on the net effect of innovation policy. A great deal of literature has taken innovation system as the research object to study the causal relationship between the two. For example, the effects of innovation-oriented city policy implementation on urban green innovation [1], industry-university-research collaborative innovation [2] and urban innovation performance [3], etc., all these studies have reached the consistent conclusion that innovation-oriented city policy implementation can significantly improve the level of innovation. In addition, some scholars have studied the impact of innovation-oriented city policies on energy efficiency [4], residents’ green lifestyle [5], etc., but they have not studied the impact of innovation-oriented city policies on entrepreneurial system. (2) Focus on the factors influencing technology entrepreneurship, such studies can be grouped into two categories. In terms of positive factors, some scholars have found that technological innovation is positively correlated with technological enterprise entrepreneurship [6]; the development of technology business incubators improves the survival rate and success rate of technology enterprises [7], but Martin came to the opposite conclusion, arguing that incubators do not create jobs [8]; and the improvement of the business environment improves the entrepreneurship of technology enterprises [9], creative personalities find business opportunities and start businesses better than others [10]. Hu and Qian found that excessively high levels of housing prices in the region would hinder individual entrepreneurial tendencies [11]. As can be seen from the above analysis, scholars have studied its impact on science and technology entrepreneurship from different perspectives, but have not yet studied its impact on science and technology entrepreneurship from the perspective of innovative city policy. (3) Focus on the overall problem of innovation and entrepreneurship. A large number of documents used principal component analysis and hierarchical analysis to measure the index of innovation and entrepreneurship [12]. Part of the literature studies regional differences in innovation and entrepreneurship. It is believed that the overall regional gap in innovation and entrepreneurship in China is gradually decreasing, and the spatial spillover effect is found to play an important role in the improvement of innovation and entrepreneurship capacity [13].

Therefore, the marginal contributions of this paper mainly include the following points: (1) Taking the innovation-type city policy implemented by the state as a quasi-natural experiment, the impact of innovation-type city policy on science and technology entrepreneurship is evaluated for the first time, which not only expands the content of innovation-type city policy research and analysis, but also enriches the current situation of science and technology entrepreneurship research system from the policy level. (2) Collect and sort out the micro-data of enterprise registration information in the enterprise check data website, and combine the detailed definition standards of science and technology enterprises in the Opinions of the Ministry of Science and Technology on Further Promoting the Innovation and Development of small and medium-sized science and technology enterprises issued by the Ministry of Science and Technology, and then describe and measure the entrepreneurial level of science and technology enterprises. Compared with previous studies, the advantage of this data is that it overcomes the single problem of enterprise type caused by the newly increased number of private enterprises in the China Statistical Yearbook, so as to measure the level of technological entrepreneurship in a more detailed and accurate way. (3) The policy spillover problem is considered, that is, the spatial spillover effect of innovation-oriented city policies on science and technology entrepreneurship is studied, and the mechanism through which such spillover effect is realized.

The second part is the theoretical mechanism and research hypothesis, which deeply analyzes the internal mechanism of innovation-oriented city policy affecting science and technology entrepreneurship. The third part is the research design; The fourth part is the empirical analysis; The fifth part is the analysis of spatial spillover effect; The sixth part is conclusion and policy enlightenment.

## 2.Theoretical mechanism and research hypothesis

### 2.1 Policy background

Innovation-oriented city policy is an important task implemented in China to improve the level of urban innovation and cultivate and develop strategic emerging industries. In 2008, with the approval of China’s National Development and Reform Commission, Shenzhen became the first innovation-oriented pilot city. In 2010, the Ministry of Science and Technology issued the Guidelines on Further Promoting the Pilot work of innovation-oriented Cities, which agreed to include 16 cities such as Dalian, Qingdao and Xiamen in the pilot work. In order to further improve the level of urban innovation and give full play to the high-end radiation and leading role of innovation-oriented cities, the Ministry of Science and Technology and the National Development and Reform Commission jointly issued the Guidelines on Building Innovation-oriented Cities in 2016, which adjusted the pilot cities in the past and finally formed a list of 61 pilot cities. In 2017, 61 pilot cities for innovation were accepted. In 2018, the two ministries further expanded the scope of the pilot cities, agreeing to Jilin City and other 17 cities into the pilot list. Up to now, 78 cities (districts) in China have implemented innovative pilot city policies.

### 2.2 The direct effect of the establishment of innovative cities on technological entrepreneurship

On the one hand, policy implementation plays a crucial role in the motivations and entrepreneurial activities of entrepreneurs [14]; on the other hand, regional innovation environments that provide support and security in innovation have a direct or indirect impact on regional entrepreneurial activities and entrepreneurial levels [15]. As a combination of policy and innovation, innovative city policies can have an important impact on the creation of technology enterprises. Specifically, innovative cities can directly increase the willingness of technology firms to start a business by: First, financial support: innovative cities require local financial institutions to increase credit support to science and technology enterprises, through the creation of science and technology banks, increase the guidance support and risk compensation for investment funds of technology enterprises, which greatly alleviate the problem of capital demand at the beginning of the enterprise. For example, after Ningbo becomes an innovative city, in order to encourage highly educated personnel from institutions of higher learning and scientific research institutions to lead and set up scientific and technological enterprises, the government at the same level will give an additional 50% subsidy on the basis of state preferential treatment; Second, property rights protection: innovative city innovation intellectual property service management, On the one hand, it has strengthened the creation, protection and application of intellectual property rights, on the other hand, it has severely punished infringements of property rights, Regulating the market order and reducing the market behavior of bad competition. To "escort" the innovation achievements of science and technology enterprises, this paper proposes the following hypothesis:

Hypothesis 1: The establishment of innovative cities improves the level of technological entrepreneurship.

### 2.3 The intermediary effect of the establishment of innovative cities on the impact of technological entrepreneurship

Studies have shown that to build innovative cities, it is necessary to increase incubator incubation threshold, incubation rate, outsourcing service function of incubator and promote interactive sharing of resources inside and outside incubation park (Bøllingtoft A) [16]. In addition, the Guidance on Further Advancing Pilot Work in Innovative Cities clearly states the need to increase the number of incubators for technology enterprises and improve the service function and professionalization of incubators. So, the creation of an innovative city is conducive to improving the technology business incubator, so how can an innovative city improve the level of technology enterprise entrepreneurship through the technology business incubator? In general, incubators, as the "cradle" of technology-based enterprises, can provide enterprises with entrepreneurial places, facility sharing, technical advice and entrepreneurial guidance, so as to reduce their entrepreneurial costs and promote their entrepreneurship [17]. For example, first, look at the "policy chain" end of the incubator. For incubators of start-up technology enterprises, tax incentives are available, while development tools and software facilities for start-up services are appropriately subsidized. Most importantly, incubators of start-up technology enterprises have free access to public R&D services and technology finance services in incubators; Second, look at the incubator’s "talent chain" end. Bring into full play the function of incubator siphon, absorb high-end talents such as returnees, foreign experts and college graduates into the incubation of entrepreneurship [18], and hire successful entrepreneurs to provide entrepreneurial guidance to incubating technology enterprises; Third, look at the Service Chain end of the incubator. Optimize and perfect the scientific and technological service platform, promote the sharing of scientific and technological resources, equipment resources and scientific and technological achievements in the incubator, and form an open entrepreneurial normal system for scientific and technological enterprises to help each other. In addition, improve the platform for cooperative communication and provide an integrated platform for the exchange of information and cooperation among the creator community.

Innovative city construction improves the level of science and technology entrepreneurship through the flow of human capital. On the one hand, innovative cities enhance the attractiveness of talents in pilot areas through policy guidance and system supply. On the other hand, innovative pilot city policies attract talent by influencing development levels in different areas of the city. For example, when a city becomes an innovative city, by increasing the level of local economic development, it affects the expected income and attracts talent [3]. So how does an innovative city improve the level of technological entrepreneurship through the flow of human capital? Due to the spillover nature of human capital [19], Individuals can not only share market information, reduce the cost of information search, but also understand industry dynamics, improve market judgment, reduce uncertainty about entrepreneurial opportunities, and increase their success rate [20]. Based on the above analysis, this paper proposes the following hypothesis:

Hypothesis 2: The establishment of innovative cities improves the entrepreneurial level of technological enterprises through the incubator of technological enterprises and the flow of human capital.

### 2.3 Spatial spillover effect of the establishment of innovative cities on the entrepreneurial impact of technological enterprises

Numerous studies have shown significant spatial spillover in regional policies [21, 22] Innovative pilot city-building has transformed urban economic activity into a whole by facilitating the movement and aggregation of various elements within the region. On the one hand, the construction of innovative pilot cities may lead to the improvement of innovation level and entrepreneurship level in surrounding cities through knowledge spillover and technology externality, thus showing positive spatial spillover effect, which means that the construction of innovative pilot cities can drive technological enterprises in surrounding cities to start businesses through "radiation effect." On the other hand, due to preferential policies and measures in the construction of innovative pilot cities, which brings together all kinds of entrepreneurial elements on a large scale within the city, Attracting large numbers of people to start businesses also means that innovative cities are promoting technological entrepreneurship at the same time, This paper proposes the following hypothesis:

Hypothesis 3: The improvement effect of innovative city construction on the entrepreneurial level of technological enterprises is spatial spillover.

## 3. Research design

### 3.1 Model settings

Based on the practice of Li et al. [23], this paper establishes the following model to study the impact of the establishment of innovative cities on the entrepreneurship of technological enterprises.

$$KJCY_{it}=\beta_{0}+\beta_{1}\times did_{it}+(S\times f(t))`\psi_{1}+X_{it}`\theta_{1}+\alpha_{i}+\lambda_{t}+\delta_{city}\times \lambda_{t}+\varepsilon_{it}$$ (1)

Compared with the traditional simple multi-time point double difference model, the above model can better meet the requirements of parallel trends and make the results more accurate because it considers the potential difference of net effect before and after policy implementation in other characteristics. Among them, KJCY_it_ is the explained variable, denoting the technology-based enterprise start-up in the i-city; The did_it_ represents an innovative city policy, of which coefficient β_1_ indicates the net effect of the establishment of an innovative city on the entrepreneurial impact of a technological enterprise. S is the selection variable for the pre-pilot as an innovative pilot city; f(t) is a primary, secondary and cubic function of time; X_it_ is a series of control variables; Fixing effects for cities; Fixed effect for years; It is a random error term for the interaction between city fixed effect and year fixed effect.

### 3.2 Variable selection and data description

#### 3.2.1 Interpreted variables

There are currently two main measures for technology entrepreneurship. The first is to use the number of businesses in the national high-tech zone to measure the level of technological entrepreneurship in the region. However, not all regions of our country have established national high-tech zones, and this method is used directly to measure the big gap in the indicators of science and technology entrepreneurship. The second is based on input and output perspective, set up evaluation index system to measure the level of science and technology entrepreneurship. However, there are many factors affecting technology entrepreneurship, and it may not be comprehensive enough to select only relevant indicators to quantify the level of technology entrepreneurship. For this reason, this article first checks the official website to select the business name, registered address, registration time, enterprise type and other information of 198.6 million newly registered enterprise data. Combined with the detailed criteria for the definition of science and technology enterprises in the "Opinions of the Ministry of Science and Technology on Further Promoting the Innovation Development of Science and Technology SMEs," issued by the Ministry of Science and Technology in 2015, Thus define technology entrepreneurship, and finally get 5638,545 new registration data of technology enterprises. The corresponding prefecture-level cities are then matched according to the place of registration and the time of registration, and finally the level of scientific and technological entrepreneurship of each prefecture-level city is obtained. The main reason for selecting the website to check the number of new enterprises is that the website covers 200 million domestic enterprise data, the total data is large and the update is fast, which can overcome the shortcomings of measuring technology entrepreneurship in the past.

#### 3.2.2 Core interpretation variable

In this paper, the policy of "Innovative Pilot City" is regarded as a quasi-natural experiment. Among them, the pilot city of implementing Innovative City Policy is set to 1, the non-pilot city is set to 0, the policy implementation year and beyond is set to 1, and the policy implementation is set to 0.

#### 3.2.3 Control variables

The control variables in this paper include: (1)level of economic development (rgdp). (2) Infrastructure (gdzc) Select fixed asset investment to indicate the level of infrastructure development in the region; (3)Financial Development (jrfz) select the per capita amount of financial institution loans to indicate the level of financial development; (4)Fiscal Expenditure (czzz) Select the value of the Gross Product Ratio (GDP) of the area covered by fiscal expenditure to indicate the level of fiscal expenditure. The required data are from the Chinese Urban Statistics Yearbook.

#### 3.2.4 Mediated variables

The intermediary variables in this paper are technology incubator and human capital flow. This article draws on the practice of Gong Bin [24] to measure the number of incubators in each prefecture-level city. Human capital flows are matched one by one using data from the China Mobile Population Dynamics Monitoring Survey (CMDS). The data required are from the China Torch Statistical Yearbook and the CMDS database of the National Health and Construction Commission.

From the descriptive statistics, we can see that the overall average and standard deviation of the level of science and technology entrepreneurship is 0.6823 and 0.2724, which indicates that the fluctuation range of the level of science and technology entrepreneurship is small during the whole sample period, which indirectly indicates that the measurement method of science and technology entrepreneurship is correct. The mean values of other control variables and mediating variables are all within a reasonable range, which further indicates that the selection of variables is correct.

## 4. Analysis of empirical results

### 4.1 baseline regression results

In this paper, the multi-phase double difference method is used to study the policy effects of innovative urban policies on the entrepreneurship of science and technology enterprises. The concrete results are shown in Table 1. The results of Table 2 show that the net effect coefficient of innovative city policy is significantly positive at the 1% level, whether it is adding control variable or not. There may be three reasons: (1) The process of building an innovative city requires the support of innovative elements such as technology, knowledge and talent, and these resources also provide the initial element needs for the start-up of a technology-based enterprise. (2) Innovative urban construction focuses on technological innovation in order to cultivate a group of innovative enterprises with independent intellectual property rights and core technologies, and this process essentially induces technological enterprises to start their own businesses. (3) Innovative cities attract technology enterprises to start their own businesses by improving technology intermediary services, upgrading technology business incubators and establishing technology banks with the goal of reducing learning costs for technology enterprises and increasing information sharing among technology enterprises.

**Table 1:** Baseline regression results.

	Model 1	Model 2	Model 3	Model 4	Model 5
did	0.3502*** (0.0000)	0.0391*** (0.0091)	0.2292*** (0.0000)	0.0021** (0.0011)	0.0320* (0.0849)
rgdp		0.3162** (0.1390)	0.2292*** (0.0000)	0.0171 (0.0111)	0.5262*** (0.0942)
gdzc			-0.3121*** (0.0860)	0.2192*** (0.0331)	0.4292*** (0.2242)
jrfz				0.0212 (0.0241)	0.0131 (0.0100)
czzc					0.0242 (0.0991)
controls×f(t)	Yes	Yes	Yes	Yes	Yes
fixation	Yes	Yes	Yes	Yes	Yes
Obs	3095	3095	3095	3095	3095
R^2^	0.1695	0.3731	0.3078	0.4423	0.5161

Note

*, **, *** indicate salience at 10%, 5% and 1%, respectively. Time, individual fixed effect and the product of control variable and f (t) are all controlled in the analysis below.

**Table 2:** Descriptive statistics of variables.

Variable	Obs	Mean value	Std	Max	Min
Kjcy	3095	0.6823	0.2724	0.7432	0.1842
rgdp	3095	3.4223	3.0345	15.2431	7.2343
gdzc	3095	0.6724	0.7923	0.9432	0.1924
jrfz	3095	0.2432	0.3934	0.6241	0.1923
czzc	3095	0.6241	0.4823	0.7234	0.1824
Kfh	3095	0.3421	0.2343	0.6341	0.2532
rlzb	3095	0.4823	0.1823	0.5234	0.1023

Regression results of control variables also fit the theoretical basis. The results of the return of economic development level show that cities with high economic development level can attract scientific and technological enterprises to start their own businesses. The impact of infrastructure on science and technology entrepreneurship is positive, indicating that the improvement of infrastructure can improve the level of science and technology entrepreneurship; Financial development has no significant effect on technology entrepreneurship; The impact of fiscal expenditure on science and technology entrepreneurship is positive, but it fails to pass the salient level. The reason is that the city fiscal expenditure is widely used and the funds used to support science and technology enterprises are relatively small, which makes the impact on science and technology entrepreneurship not significant enough.

### 4.2 robustness test

In order to ensure the robustness of the results of baseline regression, a series of robustness tests are carried out, including:

#### 4.2.1 Parallel trend test

Using the practice of Amore et al. [25], parallel trend testing is performed using event analysis and the following models are set:

$$KJ{CY}_{it}=\alpha_{0}+\prod_{k\geq -5,k\neq -1}^{5}\alpha_{k}D_{it}^{k}+X_{it}\times f(t)+\alpha_{i}+\gamma_{t}+\varepsilon_{it}$$ (2)

**Fig 1:**
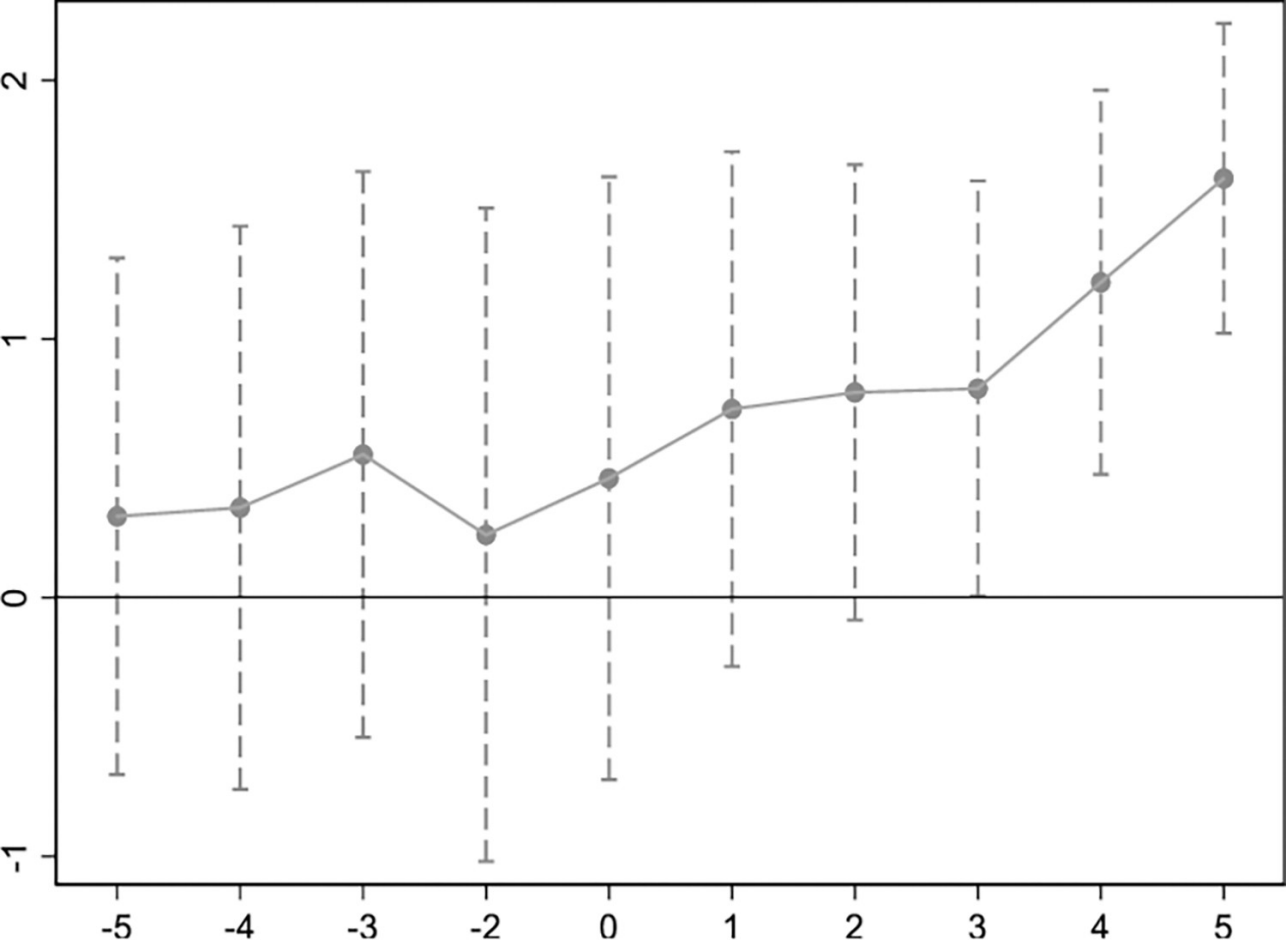
Parallel trend test.

In Formula (3), $D_{it}^{k}$ represents the dummy variable of the event of the establishment of the innovative pilot city. Suppose the establishment time of the innovative pilot city of city i is y_i,_ the K = t-y_i;_ when K≤-5, $D_{it}^{-5}$ = 1, otherwise 0; In the same way, when K = -4, -3, -2, …, 4, 5, $D_{it}^{k}$ = 1, otherwise 0; when K≥5, $D_{it}^{5}$ = 1, otherwise 0。In the specific regression analysis, this paper takes K = -1, that is, one year before the establishment of the innovation-oriented city, as the base period, so the dummy variable $D_{it}^{-1}$ is not included in Eq (3). The coefficient **α**_**k**_ that finally passes we can see if it satisfies the parallel trend test. The specific results are shown in Fig 1

The results of Fig 1 show that none of the coefficients corresponding to the four years prior to the establishment of innovative cities have passed the significance test, which means that during this period, innovative and non-innovative pilot cities had the same trend of change before the implementation of the policy, i.e. meeting the parallel trend test. In terms of dynamic effects, in the first and second years of the establishment of innovative pilot cities, although the coefficients failed the significant test, the impact on science and technology entrepreneurship has shown an increasing trend. From the third year onwards, the coefficients pass the significance test and the impact on technology entrepreneurship continues to increase. In addition, the impact of the establishment of innovative cities on entrepreneurship has shown an overall trend of improvement in terms of the value of coefficients. In summary, the improvement effect of innovative city establishment on science and technology entrepreneurship is remarkable, but there is a certain latency, and the improvement effect begins in the third year of policy implementation.

#### 4.2.2 Stabilizer inspection

**Fig 2:**
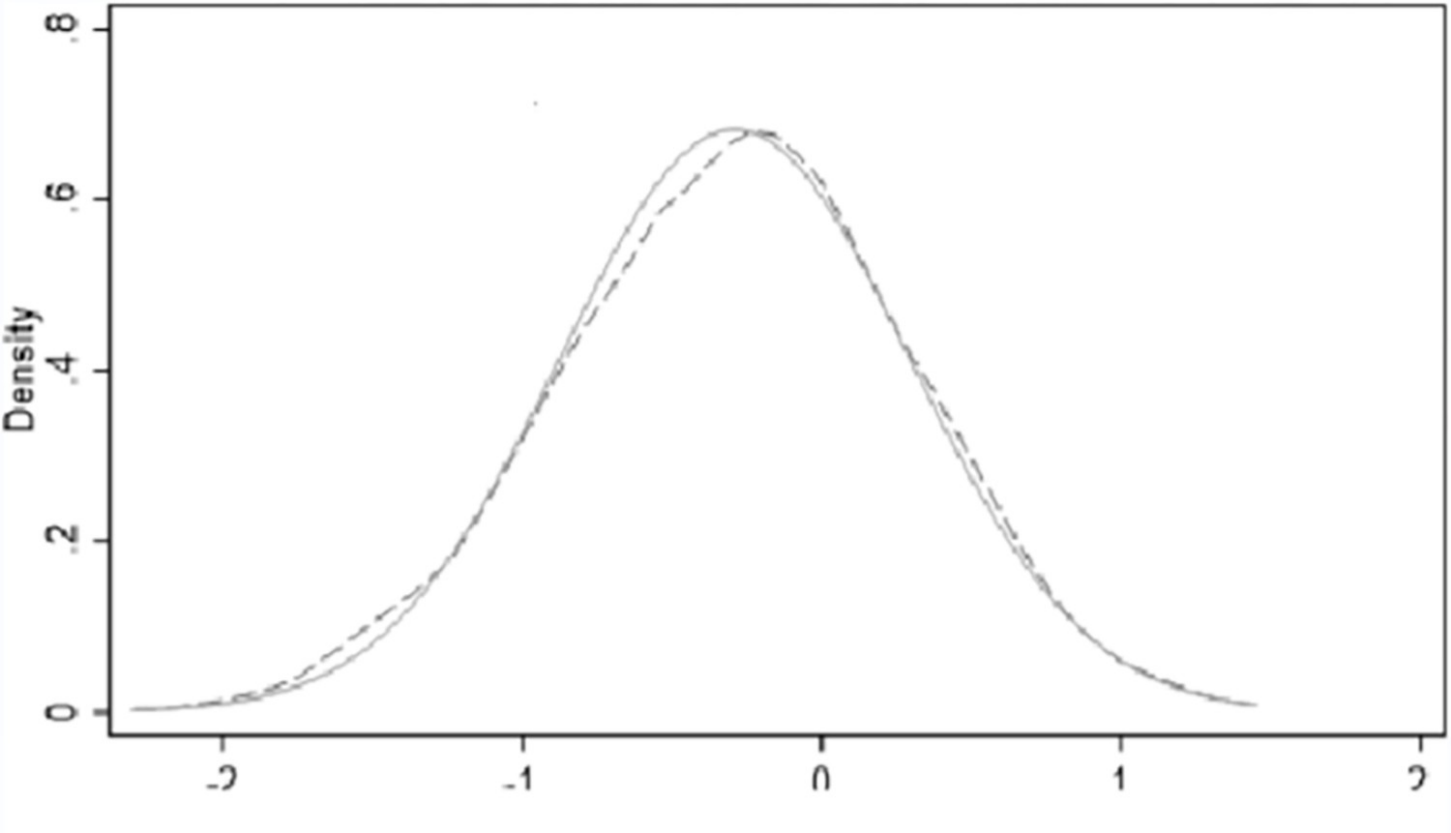
Stabilizer test.

In order to mitigate the error caused by sample selection deviation, the experimental group and control group are randomized based on Li et al. [23]. Fig 2 reports the distribution of the core density of the did coefficient in 1000 repeated experiments. The results show that the mean did coefficient is 0.0134. Very close to 0, and the coefficients obey the standard normal distribution near 0. As expected, the results of this study are robust. That is to say, the establishment of innovative cities has indeed improved the level of technological entrepreneurship.

#### 4.2.3 Other tests

(1) Exclude other policy implications. Includes the exclusion of other policy impacts implemented during sample time (commercial system reform, national entrepreneurial city construction); (2) reverse causation test. That is, whether the construction of innovative city will be affected by the reverse of scientific and technological entrepreneurship, refer to Beck et al. practice [26] to establish a risk regression model, and assume that the survival time obeys the Weible distribution, using the accelerated failure time model for regression; (3)Endogenous testing. Drawing on the practice of Zhao & Nakonieczny [27], this paper selects the product of Chinese time-honored number and time virtual variable as the tool variable of innovative urban construction and analyzes it by least squares method. When the coefficient of the tool variable is significantly positive, it indicates that the results of the base regression are robust, and vice versa. (4)Consider the robustness test of multi-point DID heterogeneous treatment effect. Baker et al. [28] found that when using the multi-point DID model for empirical analysis, there may be significant estimation bias due to the presence of a "heterogeneous processing effect." To this end, this paper uses the twowayfeweights command in STATA software to test the possible "heterogeneity" of the model, drawing on the practice of De Chaisemartin et al. [29]. The closer the result to 1, the more robust the heterogeneous test results, and the less robust the results. (5)Exclude the possibility of "moving" the control group. In the year and after the policy is implemented, if the scientific and technological enterprises in the non-pilot cities have subsidiaries in the pilot cities, then the control group can be "moved," which indicates that the level of technological entrepreneurship in the control group may also be affected by the policy shock, causing the result to deviate. In order to exclude such factors, the technology enterprises with subsidiaries in the pilot cities in and after the policy implementation in the control group will be eliminated and then reintroduced. (6)Replace tech startup metrics. In this paper, the index of science and technology entrepreneurship level is measured by the number of new enterprises, but it is measured from the quantity level, not the quality of science and technology entrepreneurship level. To this end, this paper draws on the practice of Liargovas and Repousis (2015) [30] to measure the quality of science and technology entrepreneurship by the ratio of the number of new start-ups to urban resident population. The results of the test are shown in Table 3, where the regression results indicate that the baseline estimates for Table 1 are robust.

**Table 3:** Robustness test.

	Model 6	Model 7	Model 8		Model 9	Model 10
variable	Exclude other policy influences	Reverse causality test	Endogeneity test (Stage 1)	Endogeneity test (Stage 2)	Control group movemen	New technology entrepreneurship level
did	0.0569** (0.0439)	-0.0923 (0.1792)	0.0590*** (0.0100)	0.0290*** (0.0060)	0.1482** (0.0151)	0.0756** (0.0063)
Obs	2254	3095	3095	3095	3095	2879
R^2^	0.8156	0.4654	0.6742	0.5729	0.2734	0.5237

### 4.3 heterogeneity analysis

On the basis of the above research, this paper further considers whether the impact of innovative urban construction on scientific and technological entrepreneurship is different due to the city scale, city economic development level, geographical location and new infrastructure. The following article will analyze the heterogeneity of science and technology entrepreneurship in the construction of innovative city.

#### 4.3.1 Analysis of heterogeneity of urban scale

The heterogeneity of urban scale means that large-scale cities and small-scale cities have differences in the incubator of science and technology enterprises, the flow of talents, the development of science and technology, which leads to the heterogeneity of innovative city construction to the level of science and technology entrepreneurship. This is divided by reference to the most recent standards in the State Council’s Notice on the Adjustment of Urban Size Classification Criteria, issued in 2014. According to Table 4 Model 11 and Model 12, innovative urban policies have significantly improved the level of science and technology entrepreneurship in large cities, but have not affected small-city technology entrepreneurship. Therefore, the implementation of innovative urban construction in large cities has improved the level of scientific and technological entrepreneurship, but the implementation of innovative urban construction in small cities has not produced good results. The reason is the high level of economic development in big cities, a better entrepreneurial environment and more scientific and technological resources. However, due to the relatively low level of economic development in the western region, the new infrastructure and other facilities are also poor, so that the construction of innovative cities does not play a role in promoting scientific and technological entrepreneurship.

**Table 4:** Heterogeneity analysis.

variable	Heterogeneity of city size			Heterogeneity of economic development level		Heterogeneity of location		Heterogeneity of new infrastructure	
	Big city (Model 11)		A small city (Model 12)	high (Model 13)	low (Model 14)	Central East (Model 15)	In the west (Model 16)	high (Model 17)	low (Model 18)
did	0.0062* (0.0031)	-0.0012 (0.0011)		0.3942*** (0.0900)	0.0600 (0.1871)	0.2112*** (0.0271)	-0.0181 (0.2822)	0.0031*** (0.0002)	0.0301 (0.0124)
R^2^	0.2312	0.2546		0.4326	0.3748	0.4242	0.5324	0.4235	0.3246

#### 4.3.2 Analysis of heterogeneity of economic development level

To a certain extent, the differences in economic development level represent the differences in financial subsidy, human capital level, etc., which may lead to the heterogeneity of the impact of innovative urban construction on the growth of technological entrepreneurship level. According to the average economic development level of the city, the sample is divided into areas with high economic development level and low economic development level. The results of Model 13 and Model 14 in Table 4 show that in areas with high level of economic development, the construction of innovative cities has significantly improved the level of scientific and technological entrepreneurship, but the effect of this policy is not obvious in areas with low level of economic development. The reason is that the high level of economic development area has strong scientific and technological strength, and a series of financial subsidy measures in innovation city policy can compensate for the risk cost of scientific and technological entrepreneurial activities. On the contrary, in areas with low levels of economic development, the risk costs of entrepreneurship cannot be covered, so the construction of innovative cities will not have an immediate effect on technological entrepreneurship.

#### 4.3.3 Location heterogeneity analysis

From the perspective of geographical location, the economic development level of China’s east, middle and west regions is different. Therefore, the impact of innovative city-building on technological entrepreneurship can be heterogeneous because of geographical location. To this end, this paper divides the sample cities into two parts: East Central and West. Table 4 Model 15 and Model 16 regression results show that the construction of innovative cities has significantly improved the level of scientific and technological entrepreneurship in the central and eastern regions, but it has no impact on the western regions. Because of the high level of economic development, high level of science and technology, convenient transportation, many factors that are conducive to science and technology entrepreneurship are brought together in this area, and there are many unfavorable factors in the development of science and technology entrepreneurship activities in the western region.

#### 4.3.4 Heterogeneity of new infrastructure facilities

The level of new infrastructure development represented by the Internet and big data is an important factor affecting technological entrepreneurship. In addition to the large number of entrepreneurial opportunities in areas with high levels of development of new infrastructure, there are also differences in the impact of innovative urban policies on technological entrepreneurship by providing more information support to entrepreneurs, increasing the added value of products, and thus increasing the willingness of regional scientific and technological entrepreneurship. Drawing on the practice of Feng & Wu [31], this paper uses new fixed asset investments to measure new infrastructure methods and classifies the sample of new urban fixed asset averages into areas with higher levels of new infrastructure and areas with lower levels. Table 4 Model 17 and Model 18 regression results show that, In areas with high levels of new infrastructure, innovative urban policies have significantly improved the level of technological entrepreneurship. However, policies in areas with low levels of new infrastructure have not been effective. This shows that the effect of the new urban policy implementation is related to the level of the new infrastructure.

### 4.4 Mechanism analysis

The previous empirical results show that the policy of innovative pilot cities still plays a significant role in improving the level of science and technology entrepreneurship. So, through what path are innovative pilot cities affecting the level of technological entrepreneurship in cities? Does it improve the level of technological entrepreneurship by means of technology incubator and factor flow? To verify this conjecture, this paper builds the following mediation effect model based on Wang et al. [32]:

$$MV_{it}=a_{0}+a_{1}\times did_{it}+X_{it}\times f(t)+\alpha_{i}+\lambda_{t}+\varepsilon_{it}$$ (3)

$$KJCY_{it}=b_{0}+b_{1}\times did_{it}+b_{2}MV_{it}+X_{it}\times f(t)+\alpha_{i}+\lambda_{t}+\varepsilon_{it}$$ (4)

Where MVit is an intermediary variable. b1 and b2 are the regression coefficients of innovative cities and intermediary variables, respectively. Specific regression results are shown in Table 5.

**Table 5:** Mechanism analysis.

variable	Technology Business Incubator		Human capital flow	
	Model 19	Model 20	Model 21	Model 22
did	0.1764*** (0.0366)		0.1451*** (0.0389)	
Intermediate variable		0.0701*** (2.9100)		0.1053*** (0.0297)
Sobel test	0.1119 (p = 0.0000)		0.1289 (p = 0.0000)	
Bootstrap test (Indirect effect)	0.1119 (p = 0.0000)		0.1289 (p = 0.0000)	
Bootstrap test (Direct effect)	0.1092 (p = 0.0000)		0.1009 (p = 0.0000)	
R^2^	0.2312	0.3253	0.5243	0.3245

The regression results of technology incubator as intermediary variable are shown in Table 5 Model 19 and Model 20. Model 19 shows that the impact coefficient of new urban construction on the incubator of science and technology enterprises is 0.1764, which is significant at 1% level. In order to better improve the level of urban innovation and better serve mass entrepreneurship, innovative urban policies need to increase the number of incubators for technology enterprises. Model 20 shows that the estimated coefficient of the technology business incubator is 0.0701 and is significant at 1% level, indicating that the technology business incubator will improve the level of the technology entrepreneurship, this is consistent with the research conclusion of Sansone et al. (2020) [7], indicating that technology business incubators have a positive impact on technology entrepreneurship. The Technology Business Incubator can provide physical space and infrastructure, provide numerous service support, thereby reducing the entrepreneurial risk and cost of the technology business and increasing the success rate of the technology business.

The regression results of human capital flow as intermediary variable are shown in Table 5 Model 21 to Model 22. Model 21 shows that the influence coefficient of innovative city establishment on human capital flow is 0.1451, which is significant at 1% level. In order to strengthen the cultivation of talents, implement the strategy of strengthening the country of talents in depth and increase the proportion of scientific and technological personnel, the construction of innovative cities will siphon the people in the surrounding areas and promote the orderly movement of personnel. Model 22 shows that the estimated coefficient of human capital flow is 0.1053 and is significant at 10% level, indicating that human capital flow increases the level of technological entrepreneurship. This is because human capital flows can facilitate information exchange, and entrepreneurs can remove favorable information from them, improve market judgment, reduce entrepreneurial cost investment, and thus increase the likelihood of scientific and technological entrepreneurial activities. The above results show that innovative urban construction improves the level of science and technology entrepreneurship through the flow of human capital.

In order to test the robustness of the above results, this paper uses Sobel method and bootstrap method to test the incubator and factor flow mediation effect. As can be seen from Table 5, both the incubator and the flow of human capital, the direct and indirect effects are large and significant. It also shows that technology incubator and human capital flow have a real intermediary effect on the causal relationship between innovative cities and technology entrepreneurship.

### 4.5 Spatial spillover effect analysis

The spillover of regional policy has been recognized by many scholars [21, 22] and when cities develop to a certain extent, they have an "echo effect" and a "diffusion effect" on surrounding cities. We know from the forefront that the implementation of innovative urban policies can significantly improve the level of technological entrepreneurship in our cities. But the impact on the level of technology entrepreneurship in neighboring cities is uncertain. It is impossible to determine whether there is a "radiation effect" and a "siphon effect" on the impact of the creation of an innovative city on the surrounding city. (SDID) Study the spillover of innovative urban policies, as follows:

$$KJCY_{it}=\beta_{0}+\mu WKJCY_{it}+\beta_{1}\times did_{it}+X_{it}\times f(t)+kWdid_{it}+\eta WX_{it}+\alpha_{i}+\lambda_{t}+\varepsilon_{it}$$ (5)

Among them, the μ is the spatial lag coefficient of the explained variable, the β1 is to establish the influence of the technological enterprise entrepreneurship for the innovative city, k is the influence coefficient of the innovative pilot city and W is the spatial weight matrix. In this paper, two kinds of weight matrices are constructed, the first is the geographic distance spatial weight matrix, $W_{ij}^{g}=1/d_{ij}^{2}$ (当i≠j),instead, 0, where dij is calculated by the latitude and longitude of each city; The second is the economic distance space weight matrix, $W_{ij}^{e}=W_{ij}^{g}\times \mathrm{diag}\left(\frac{\bar{Y_{1}}}{Y},\frac{\bar{Y_{2}}}{Y},\ldots ,\frac{\bar{Y_{N}}}{Y}\right),\;\bar{Y}$ is the average per capita GDP of the city and Y is the average per capita GDP of the total sample during the sample period.

#### 4.5.1 Spatial spillover effect and decomposition

From Table 6 overall, the effect coefficient of siphon effect of innovative city construction on science and technology entrepreneurship is significantly positive at 1% level, but the effect coefficient of radiation effect on science and technology entrepreneurship is positive but not significant. This indicates that the innovation cities have significantly improved the level of technological entrepreneurship in the region through the siphon effect. This is mainly due to the high risk and long economic return cycle of the start-up activities of the technology enterprises, which requires that the capital chain of the technology enterprises cannot be broken, and the areas where innovative pilot cities have been implemented can solve this problem. The implementation of innovative pilot cities requires all kinds of financial institutions to solve the problem of financing difficulties and expensive financing of science and technology enterprises, strengthen loan support and credit support for science and technology enterprises, and accelerate the gathering of social capital to science and technology enterprises. This is also the case, attracting people from other regions to start their own businesses in innovative urban areas.

**Table 6:** Spatial spillover effects at different weights.

variable	Matrix of geography		Economic matrix	
	Effect of radiation	Effect of siphon	Effect of radiation	Effect of siphon
did	0.0059 (0.1000)	0.0581*** (2.6110)	0.0456 (0.7101)	0.0121*** (2.6900)
Log-L	3791.2430		4037.6751	
R^2^	0.4237	0.3893	0.5231	0.4478

In order to learn more about the effect of spatial spillover effect, this paper draws on the practice of Elhorst [33] to partial the spatial spillover effect of SDID. The effect decomposition results under different weight matrices are shown in Table 7. In general, the siphon effect is greater than the radiation effect under different matrices. This is because science and technology entrepreneurs are attracted to local entrepreneurship by a range of incentives implemented in the innovative pilot cities region, namely, improving the level of regional technology entrepreneurship through the siphon effect. This also means that the implementation of innovative urban policies has not played a high-end role in raising the level of technological entrepreneurship in other regions.

**Table 7:** Spatial effect decomposition.

variable	Effect of radiation		Effect of siphon	
	Matrix of geography	Economic matrix	Matrix of geography	Economic matrix
did	0.0277 (0.5110)	0.0013* (1.8800)	0.0877** (2.4991)	0.0391* (0.5611)

#### 4.5.2 Spatial spillover effect mechanism test

In order to verify whether an innovative city plays a siphon effect on technological entrepreneurship through the incubator of technological enterprises or through the flow of human capital, the above two intermediary variables are regressed as explained variables. The specific regression results are shown in Table 8.

**Table 8:** Spatial overflow mechanism test.

variable	Matrix of geography		Economic matrix	
	Model 23	Model 24	Model 25	Model 26
fsdid	0.0051** (0.0020)	0.0270 (0.1531)	0.0151** (0.0381)	0.0081 (0.0495)
hxdid	0.0352** (0.0241)	0.0243 (0.4610)	0.0569*** (0.0000)	0.0103*** (0.0046)
Log-L	6532.3534	2357.4741	3345.6231	3289.3653
R^2^	0.2341	0.4123	0.3273	0.2922

Model 23 and Model 25 are the regression results of the technology business incubator under the geographic matrix and the economic matrix respectively. The regression results show that the radiation estimation coefficients of the incubator are 0.0051 and 0.0151, And they are all at the 5% level, which shows that innovative cities increase the number of incubators in technology enterprises through radiation effects. While the estimated coefficients by siphoning effect are 0.0352 and 0.0569, The results show that innovative city construction increases the number of incubators by siphon effect. The explanation is that the construction of an innovative city promotes the sharing of resources inside and outside the incubation park, which can provide enterprises with entrepreneurial space, sharing measures, technical services and entrepreneurial services.

Model 24 and model 26 are the regression results of human capital flow under corresponding weights. The estimation coefficient of factor flow for innovative pilot cities in model 24 is 0.0270, but it is not significant, and the estimation coefficient of model 26 is 0.0081. Under the geographical matrix, innovative pilot cities did not significantly improve the level of science and technology entrepreneurship through the siphon effect. Under the economic matrix, the estimated coefficient was 0.0103, which shows that after considering economic factors and innovation policy factors, innovative pilot cities improved the level of science and technology entrepreneurship significantly through the siphon effect.

## 5. Conclusions and recommendations

Using the policy of innovative pilot cities implemented in China as a quasi-natural experiment, the empirical test of the influence of the establishment of innovative cities on the level of science and technology entrepreneurship through the construction of multi-time point double difference model is conducive to separating the net effect of innovative cities on the level of science and technology entrepreneurship from other unobservable factors. In this paper, the data of prefecture-level city panels in China from 2008 to 2020 are selected as the study samples. (1) The implementation of innovative urban policy can significantly improve the level of science and technology entrepreneurship, but the pilot policy has a time lag effect and has a continuous promoting effect since the third year. (2) For large cities, areas with high levels of economic development, central and eastern regions and cities with high new infrastructure, innovative cities can improve the level of technological entrepreneurship; (3) Innovative cities increase the level of urban entrepreneurship by improving incubators for technological enterprises and strengthening the flow of human capital; (4) The effect of the establishment of innovative cities on the level of technological entrepreneurship mainly shows that the siphon effect does not show radiation effect, and this siphon effect mainly improves the level of technological entrepreneurship by increasing the number of incubators of technological enterprises.

Based on the above conclusions, this paper puts forward the following suggestions: (1) A new urban development model marked by innovative urban construction plays an important role in improving the level of science and technology entrepreneurship. Therefore, we should persist in promoting innovative pilot cities in a step-by-step manner, forming replicable outreach experiences and expanding policy reach. It is also necessary to continuously increase the number of incubators and the flow of human capital to improve the level of technological entrepreneurship. In addition, it is also necessary to further improve the innovation-oriented city policy system, strengthen the supervision and evaluation mechanism, refine the city selection conditions, strengthen supervision and feedback, and ensure the smooth implementation of policies. (2) Considering the policy lag, the Government should gradually deepen the implementation of innovative urban policies at different times. Given the heterogeneous effects of policy implementation, policies should be formulated to avoid "one size fits all" behaviour and targeted implementation of innovative urban policies. In addition, in advancing the work of innovative pilot cities, the government should not only give full play to the role of the "leader" of entrepreneurship in large cities and areas with high levels of economic development, but also strengthen the level of scientific and technological entrepreneurship in western regions and areas with weak new infrastructure. Therefore, in the future innovative city policy pilot process, it is necessary to strengthen the overall planning and coordinated development among different cities, formulate and implement relevant policies according to local conditions, and promote the coordinated development of urban technological entrepreneurship level. More importantly, in the future process of economic development, the government should further strengthen the policy design of western regions, give full play to the driving role of policies, and better promote the coordinated development of regional economy. (3) Play the role of siphon in innovative cities and strengthen the role of demonstration in central cities. In the process of innovative pilot city construction, we can increase the number of incubators of science and technology enterprises, and then generate spatial spillover. Therefore, effective use of innovative city policy siphon is undoubtedly the key to improving the level of science and technology entrepreneurship.

This paper evaluates the impact of innovation-oriented city policies on science and technology entrepreneurship from both theoretical and empirical aspects, and provides a policy reference for subsequent countries to better improve the level of science and technology entrepreneurship. Since this paper focuses on the level of science and technology entrepreneurship, the quality of science and technology entrepreneurship is not studied in detail. It only uses the quality of science and technology entrepreneurship as a replacement index in the robustness test, but does not carry out in-depth analysis. Therefore, in future research, it is a very good direction to conduct detailed research on the quality of science and technology entrepreneurship from the growth performance of new ventures.

## References

[1] Li L, Li M, Ma S, et al. Does the construction of innovative cities promote urban green innovation?[J]. Journal of Environmental Management, 2022, 318: 115605. 35759959 10.1016/j.jenvman.2022.115605

[2] Zhang S, Wang X, Zhang B. The policy effects of innovative city pilot on the dual efficiency of industry–university–research knowledge flow[J]. Technology Analysis & Strategic Management, 2022, 34(9): 1038–1049.

[3] Gao K, Yuan Y. Government intervention, spillover effect and urban innovation performance: Empirical evidence from national innovative city pilot policy in China[J]. Technology in Society, 2022, 70: 102035.

[4] Yu Y, Chen X, Zhang N. Innovation and energy productivity: An empirical study of the innovative city pilot policy in China✰[J]. Technological Forecasting and Social Change, 2022, 176: 121430.

[5] Zhang J, Zheng T. Can dual pilot policy of innovative city and low carbon city promote green lifestyle transformation of residents?[J]. Journal of Cleaner Production, 2023: 136711.

[6] Fang Z, Razzaq A, Mohsin M, et al. Spatial spillovers and threshold effects of internet development and entrepreneurship on green innovation efficiency in China[J]. Technology in Society, 2022, 68: 101844.

[7] Sansone G, Andreotti P, Colombelli A, et al. Are social incubators different from other incubators? Evidence from Italy[J]. Technological Forecasting and Social Change, 2020, 158: 120132.

[8] Lukeš M, Longo M C, Zouhar J. Do business incubators really enhance entrepreneurial growth? Evidence from a large sample of innovative Italian start-ups[J]. Technovation, 2019, 82: 25–34.

[9] Wei Y. Regional governments and opportunity entrepreneurship in underdeveloped institutional environments: An entrepreneurial ecosystem perspective[J]. Research Policy, 2022, 51(1): 104380.

[10] Shane S, Nicolaou N. Creative personality, opportunity recognition and the tendency to start businesses: A study of their genetic predispositions[J]. Journal of Business Venturing, 2015, 30(3): 407–419.

[11] Hu F Z Y, Qian J. The impact of housing price on entrepreneurship in Chinese cities: Does the start-up motivation matter?[J]. Cities, 2022: 104045.

[12] Bin Gao, Xinxing Duan. Construction and measurement of provincial innovation and entrepreneurship environment evaluation index system [J]. Statistics and Decision, 201,37(12):70–73.(in Chinese)

[13] LIU C M. Spatial differentiation and convergence of China’s innovation and entrepreneurship capacity. China Population Science,2022(02):99–111+128.

[14] Fonseca R, Lopez-Garcia P, Pissarides C A. Entrepreneurship, start-up costs and employment[J]. European Economic Review.2001,45(4–6): 692–705.

[15] Beugelsdijk S, Noorderhaven N. Entrepreneurial attitude and economic growth: A cross-section of 54 regions[J]. The Annals of Regional Science, 2004, 38(2): 199–218.

[16] Bøllingtoft A. The bottom-up business incubator: Leverage to networking and cooperation practices in a self-generated, entrepreneurial-enabled environment[J]. Technovation, 2012, 32(5): 304–315.

[17] Al-Awlaqi M A, Aamer A M, Habtoor N. The effect of entrepreneurship training on entrepreneurial orientation: Evidence from a regression discontinuity design on micro-sized businesses[J]. The International Journal of Management Education, 2021, 19(1): 100267.

[18] Meyer M. Domesticating and democratizing science: a geography of do-it-yourself biology[J]. Journal of Material Culture, 2013, 18(2): 117–134.

[19] Lucas R E. Jr On the mechanics of economic development[J]. Journal of monetary economics.1988,22(1): 3–42.

[20] Zheng S, Du R. How does urban agglomeration integration promote entrepreneurship in China? Evidence from regional human capital spillovers and market integration[J]. Cities, 2020, 97: 102529.

[21] Li S, Liu J, Wu J, et al. Spatial spillover effect of carbon emission trading policy on carbon emission reduction: Empirical data from transport industry in China[J]. Journal of Cleaner Production, 2022, 371: 133529.

[22] Li Z, Wang J. Spatial spillover effect of carbon emission trading on carbon emission reduction: Empirical data from pilot regions in China[J]. Energy, 2022, 251: 123906.

[23] Li P, Lu Y, Wang J. Does flattening government improve economic performance? Evidence from China[J]. Journal of Development Economics.2016,123: 18–37.

[24] Bin Gong. How Science and Technology Business Incubator activates Regional innovation—The mediating role of venture capital and incubation Fund [J]. Science and Technology Progress and Countermeasures, 201,38(01):34–44.

[25] Amore M D, Schneider C, Žaldokas A. Credit supply and corporate innovation[J]. Journal of Financial Economics.2013,109(3): 835–855.

[26] Beck T, Levine R, Levkov A. Big bad banks? The winners and losers from bank deregulation in the United States[J]. The Journal of Finance.2010,65(5): 1637–1667.

[27] Zhao X, Nakonieczny J, Jabeen F, et al. Does green innovation induce green total factor productivity? Novel findings from Chinese city level data[J]. Technological Forecasting and Social Change, 2022, 185: 122021.

[28] Baker A C, Larcker D F, Wang C C Y. How much should we trust staggered difference-in-differences estimates?[J]. Journal of Financial Economics.2022,144(2): 370–395.

[29] De Chaisemartin C, d’Haultfoeuille X. Two-way fixed effects estimators with heterogeneous treatment effects[J]. American Economic Review.2020,110(9): 2964–96.

[30] Liargovas P, Repousis S. Development paths in the knowledge economy: innovation and entrepreneurship in Greece[J]. Journal of the Knowledge Economy, 2015, 6: 1063–1077.

[31] Feng Q, Wu G L. On the reverse causality between output and infrastructure: The case of China[J]. Economic Modelling, 2018, 74: 97–104. doi: 10.1016/j.econmod.2018.05.006

[32] Wang M, Huang Y, An Z, et al. Reforming the world’s largest heating system: Quasi-experimental evidence from China[J]. Energy Economics,2022,106417. doi: 10.1016/j.eneco.2022.106417

[33] Elhorst J P. Applied spatial econometrics: raising the bar[J]. Spatial economic analysis.2010,5(1): 9–28.

